# Neutrophil Cathepsin G Enhances Thrombogenicity of Mildly Injured Arteries via ADP-Mediated Platelet Sensitization

**DOI:** 10.3390/ijms23020744

**Published:** 2022-01-11

**Authors:** Abderrahim Nemmar, Marc F. Hoylaerts

**Affiliations:** 1 Department of Physiology, College of Medicine and Health Sciences, United Arab Emirates University, Al-Ain P.O. Box 17666, United Arab Emirates; anemmar@uaeu.ac.ae; 2 Center for Molecular and Vascular Biology, Department of Cardiovascular Sciences, University of Leuven, 3000 Leuven, Belgium

**Keywords:** neutrophils, neutrophil extracellular traps, protease activated receptors, P2 receptors, lung inflammation, thrombosis, erythrocytes, air pollution, particulate matter

## Abstract

Inhalation of particulate matter in polluted air causes direct, size-restricted passage in the circulation and pronounced lung inflammation, provoking platelet activation and (non)-fatal cardiovascular complications. To determine potency and mechanism of platelet sensitization via neutrophil enzymes, we performed in vitro aggregation studies in washed human platelets and in murine and human blood, in the presence of elastase, cathepsin G and regular platelet agonists, present in damaged arteries. The impact of both enzymes on in vivo thrombogenicity was studied in the same thrombosis mouse model, previously having demonstrated that neutrophil activation enhances peripheral thrombogenicity. At 0.05 U/mL, cathepsin G activated washed human platelets via PAR1, whereas at 0.35 U/mL, aggregation occurred via PAR4. In Swiss mouse platelet-rich plasma no aggregation occurred by cathepsin G at 0.4 U/mL. In human and murine blood, aggregations by 0.05–0.1 U/mL cathepsin G were similar and not PAR-mediated, but platelet aggregation was inhibited by ADP antagonists, advocating cathepsin G-released ADP in blood as the true agonist of sustained platelet activation. In the mouse thrombosis model, cathepsin G and elastase amplified mild thrombogenicity at blood concentrations that activated platelets in vitro. This study shows that cathepsin G and elastase secreted in the circulation during mild air pollution-induced lung inflammation lyse red blood cell membrane proteins, leading to ADP-leakage into plasma, sensitizing platelets and amplifying their contribution to cardiovascular complications of ambient particle inhalation.

## 1. Introduction

Exposure to particulate matter in polluted air for hours to weeks triggers cardiovascular disease-related mortality and non-fatal events, in conjunction with arterial thrombotic complications [1,2,3]. Long-term exposure over years further increases the risk of cardiovascular mortality, as a result of vascular degenerative disease, caused by chronic inflammation, oxidative stress and atherosclerosis [1,4,5]. Experimental studies in animal models have reproduced the inhalation of particulate matter via intratracheal (i.t.) instillation and have demonstrated that instillation of a dose of diesel exhaust particles (DEP), representative of a 24 h inhalation interval in man causes rapid platelet activation, in turn eliciting arterial thrombogenicity of compromised arterial blood vessels [6]. Furthermore, detailed studies in man and hamsters showed that the rapid sensitization of circulating platelets was due to the direct translocation of inhaled ultrafine particles into the blood stream within 1 h [6,7,8]. However, i.t. instilled particulate matter in the hamster also produced lung inflammation over an interval of 6–24 h, resulting in neutrophil activation and histamine release in the circulation, promoting lung infiltration of neutrophils, a process largely interrupted by histamine antagonism [9]. Histamine antagonism also reduced arterial thrombogenicity, especially in the same 6–24 h time interval, linking lung inflammation and circulating platelet sensitization.

The direct interaction of ultrafine particles (particles with a diameter < 100 nm) with platelets causes platelet activation [6], a process which is partly mediated by the positive charges present on ultrafine particles [10], but the downstream consequences of neutrophil activation by air pollutants for subsequent platelet sensitization have never been clarified in detail. Whereas platelets facilitate the docking of leukocytes on the endothelium [11,12] and platelet-leukocyte heteroconjugates are enhancing the particulate matter-triggered prothrombotic tendency [13], it remains elusive whether platelets are activated by neutrophils primarily via cell–cell contact or whether peripheral thrombogenicity on small vascular fissures (present in and on compromised coronary blood vessels) is enhanced via neutrophil-released proteases.

Several serine proteases, such as mast cell tryptase, trypsin, plasmin and cathepsin G, are capable of activating platelets [14], with platelet activation by plasmin having been coupled to the secretion of von Willebrand factor [15]. All these enzymes are reported to proteolytically activate platelet PAR4 [16,17,18]. Activated polymorphonuclear leukocytes (PMNs) release three serine proteases: non-specific proteinase 3, trypsin-like elastase and chymotrypsin-like cathepsin G [19]. Whereas cathepsin G is a strong platelet agonist [20], elastase has not been recognized as such, although it seems to proteolytically activate the platelet integrin αIIbβ3 through cleavage of the carboxyl terminus of the αIIb subunit heavy chain [21]. Neutrophil elastase potentiates the cathepsin G-induced platelet activation [22]. During sepsis, but also during normal blood coagulation, these proteases can be detected in the circulation in high concentrations even when in plasma, their activity is limited by several proteinase inhibitors [23], but aggregates between platelets and PMNs, activated PMNs and neutrophil extracellular traps (NETs) can be found at sites of vascular injury and inflammation, possibly in microenvironments, where cathepsin G is protected from proteinase inhibitors [23,24]. Cathepsin G strongly resembles thrombin in its ability to activate human platelets, be it that it activates platelets predominantly via PAR-4 cleavage [25] Cathepsin G cleaves PAR1 at the N-terminal thrombin cleavage site, but inactivates the receptor via a second downstream cleavage site [26]. Like thrombin, cathepsin G stimulates the phospholipase C/protein kinase C pathway, required for platelet functionality via PAR4 [27].

Because cathepsin G has been uncovered as an agent implicated in experimental thrombosis [28], we have presently investigated whether and how low levels of cathepsin G and elastase can sensitize/activate resting platelets. Furthermore, to validate cooperation between low cathepsin G levels and low concentrations of platelet agonists, present during mild vascular lesions, we performed a series of mixed in vitro aggregation assays in human and in murine platelets. We also made use of the same experimental animal thrombosis model, applied before in our air pollutant studies [9], to validate the impact of infused cathepsin G and elastase on peripheral thrombogenicity, upon inflicting mild vascular injury. These studies revealed that circulating cathepsin G releases ADP from red blood cells (RBCs), in concentrations sufficiently high to sensitize platelets via ADP receptors, priming them for thrombotic responses to mild vascular injury, independently of PAR1 and PAR4.

## 2. Results

### 2.1. Cathepsin G and Elastase Threshold Concentration Definition

**Figure 1:**
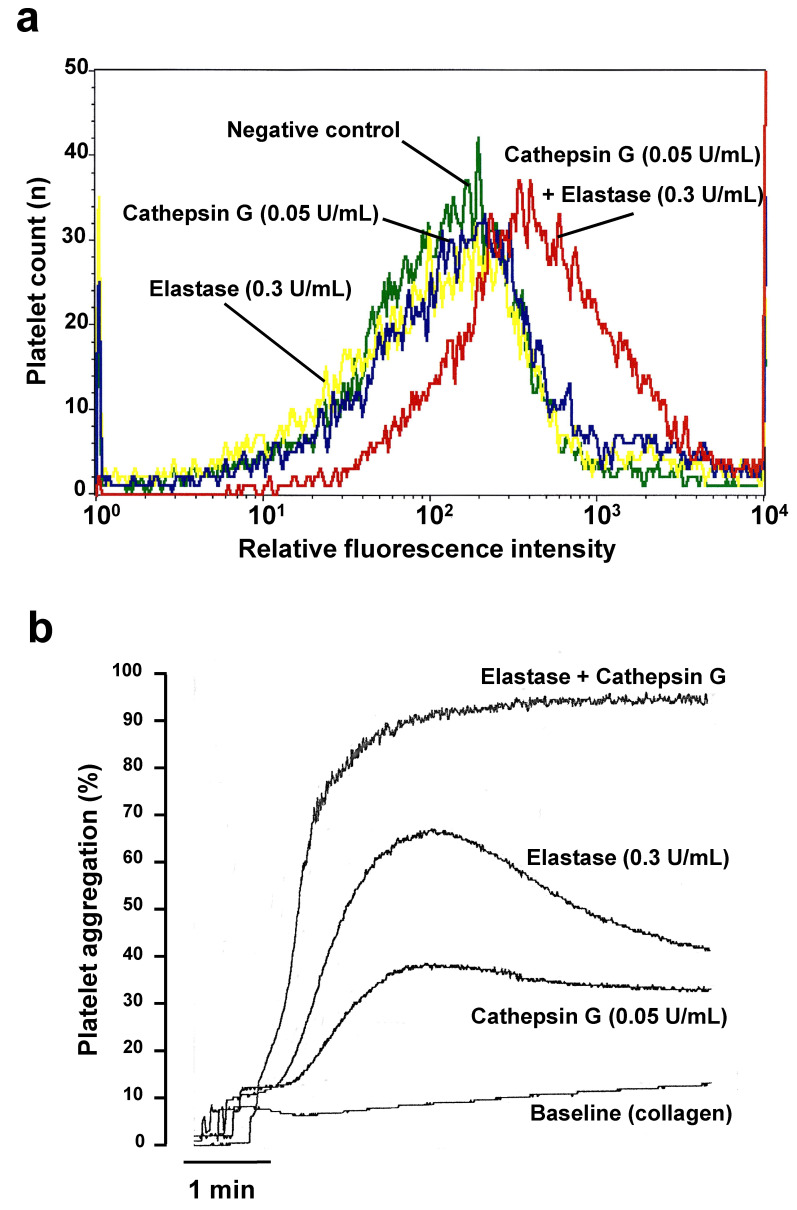
Synergistic effect of low cathepsin G and elastase concentrations on human platelet activation. (**a**) Relative fluorescence histograms for the distribution of activated washed platelets expressing P-selectin (CD62P), in non-activated controls (green line) and after static activation for 6 min at 37 °C with 0.05 U/mL neutrophil cathepsin G (blue line), 0.3 U/mL elastase (yellow line), or their combination (red line). (**b**) Aggregation tracings for washed human platelets by the threshold collagen concentration (Baseline, 0.1 µg/mL), with or without platelet pretreatment by 0.05 U/mL cathepsin G, 0.3 U/mL elastase or their combination for 6 min at 37 °C, as specified. Continuous tracings recorded on a Chrono-log Aggregometer are representative of three to five independent experiments, performed on platelets from different individuals.

To define the minimal concentration of cathepsin G and elastase, capable of sensitizing platelets, we first incubated washed human platelets under static conditions with various concentrations of cathepsin G and/or elastase. Via flow cytometry detection of platelet surface P-selectin, Figure 1a shows that 0.05 U/mL cathepsin G cannot activate platelets distinctly, in the absence of other platelet agonists. Likewise, 0.3 U/mL elastase alone did not induce distinct platelet P-selectin expression in isolated platelets. However, when platelets were incubated with both PMN-derived enzymes simultaneously, the CD62P positive platelet numbers increased, confirming that the minimal agonist activity of cathepsin G on platelets can be enhanced by added elastase, even when elastase is a poor platelet agonist itself. Correspondingly, the aggregation of washed human platelets by threshold collagen concentrations, themselves not capable of triggering measurable platelet aggregation (Baseline in Figure 1b) was stimulated by both elastase (0.3 U/mL) and cathepsin G (0.05 U/mL), and, in agreement with Figure 1a, the combined addition of both enzymes enhanced platelet aggregation synergistically, potently and irreversibly over a few minutes (Figure 1b).

### 2.2. Role of PAR1 and PAR4 in Washed Human Platelet Activation by Cathepsin G

**Figure 2:**
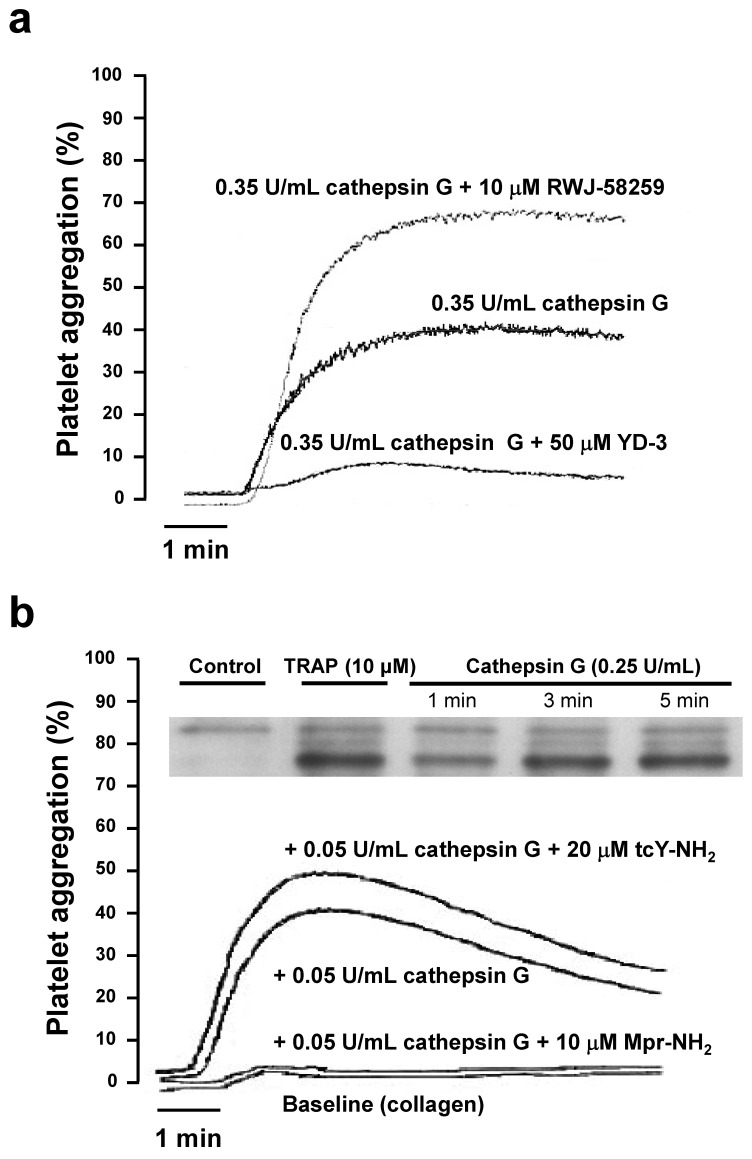
Washed human platelet aggregation: Role of PAR1 and PAR4. (**a**) Washed human platelet aggregation tracings by 0.35 U/mL cathepsin G, in the absence or presence of the PAR1 antagonist RWJ-58259 or the PAR4 antagonist YD-3, at the indicated concentrations. (**b**) Washed human platelet aggregation tracings by the threshold collagen concentration (Baseline, 0.1 µg/mL) alone, or after the additional platelet pretreatment for 6 min at 37 °C with 0.05 U/mL cathepsin G, in the absence or presence of the PAR1 antagonists Mpr-NH_2_ or the PAR4 antagonist tcY-NH_2_ at the indicated concentrations. Insert: Western blot showing phospholipase Cβ2 phosphorylation by TRAP and by cathepsin G at the specified time and concentrations. Continuous tracings recorded on a Chrono-log aggregometer are representative of 3–5 independent experiments, performed on platelets from different individuals.

In agreement with previous characterization [25], at higher cathepsin G concentrations (0.35 U/mL), human platelet aggregation was substantial and almost entirely PAR4 mediated (i.e., was strongly inhibited in the presence of the PAR4 antagonist YD-3). The PAR1 antagonist even stimulated platelet activation (Figure 2a), which may be related to PAR1-PAR4 heterodimerization [29], a phenomenon which was not further investigated in this study. 

This part confirms that washed platelet activation occurs predominantly via PAR4, at high neutrophil cathepsin G concentrations. Platelet activation by high cathepsin G concentrations (0.25 U/mL) triggered phospholipase Cβ2 phosphorylation (Figure 2b, insert), comparable to that observed with activation by Thrombin Receptor Activation Peptide (TRAP). However, when combined with threshold concentrations of collagen, low cathepsin G concentrations (0.05 U/mL) activate washed human platelets via PAR1, a profile abrogated in the presence of the PAR1 antagonist Mpr-NH_2_ (Figure 2b). The absence of any impact on the aggregation by the PAR4 antagonist tcY-NH_2_ confirmed that at low cathepsin G concentrations, washed platelet activation occurred exclusively via PAR1.

### 2.3. Human whole Blood Platelet Aggregation

**Figure 3:**
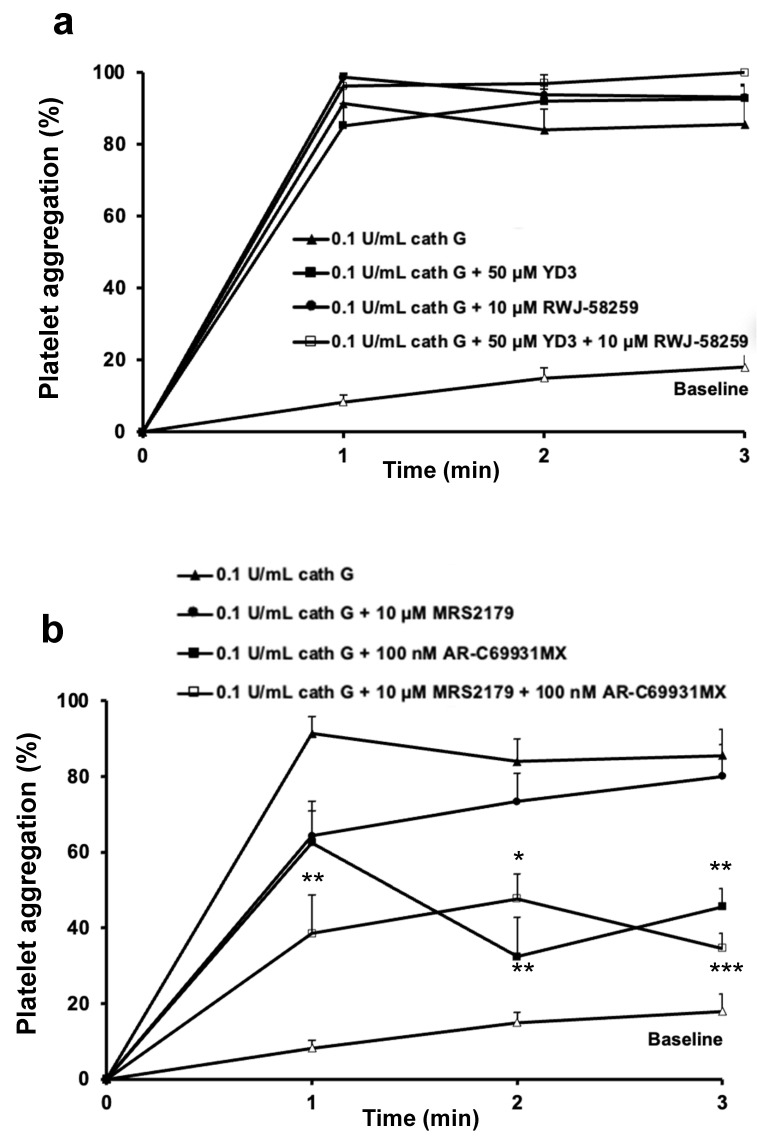
Human whole blood aggregation: role of ADP. (**a**) Platelet aggregation curves in human blood, without agonist (Baseline, open triangles), or upon addition of 0.1 U/mL cathepsin G (cath G), in the absence or the presence of the PAR1 antagonist RWJ-58259, the PAR4 antagonist YD-3 or their combination, at the concentrations indicated in the insert. (**b**) Platelet aggregation curves in human blood, without agonist (Baseline, open triangles), or upon addition of 0.1 U/mL cathepsin G (cath G), in the absence or the presence of the P2Y_1_ and P2Y_12_ receptor antagonists MRS2179 and AR-C69931MX, respectively, or their combination, at the concentrations indicated in the insert. Data are mean ± SEM (*n* = 3 in each group). Statistical analysis was by one-way ANOVA, followed by Newman–Keuls multiple-comparison test (* *p* < 0.05, ** *p* < 0.01, *** *p* < 0.001 vs. tracing for 0.1 U/mL cathepsin G). Platelet aggregation is calculated from residual platelet numbers at the indicated time points.

When platelet aggregation was studied in whole blood via a counting technique, cathepsin G (0.1 U/mL) by itself triggered complete platelet aggregation (Figure 3a). 

In contrast to the results in washed platelets (Figure 2b), neither the PAR-1 antagonist RWJ-58259 nor the PAR-4 antagonist YD-3 had any impact on the cathepsin G mediated platelet aggregation, nor did their combination (Figure 3a). On the contrary, whole blood aggregation was weakly inhibited by the P2Y_1_ antagonist MRS2179 (10 µM), only slowing down the initial rate of the cathepsin G mediated platelet aggregation after 1 min. In addition, the P2Y_12_ receptor antagonist AR-C69931MX (100 nM) reduced platelet aggregation by 55% after 2 and 3 min, whereas both antagonists combined reduced aggregation by 60% over the entire interval (Figure 3b). These experiments identified ADP, liberated by cathepsin G as the intermediate agonist activating platelets in whole blood, at the expense of PAR receptors.

### 2.4. Platelet Aggregation in Murine Platelet-Rich Plasma and in Whole Blood

**Figure 4:**
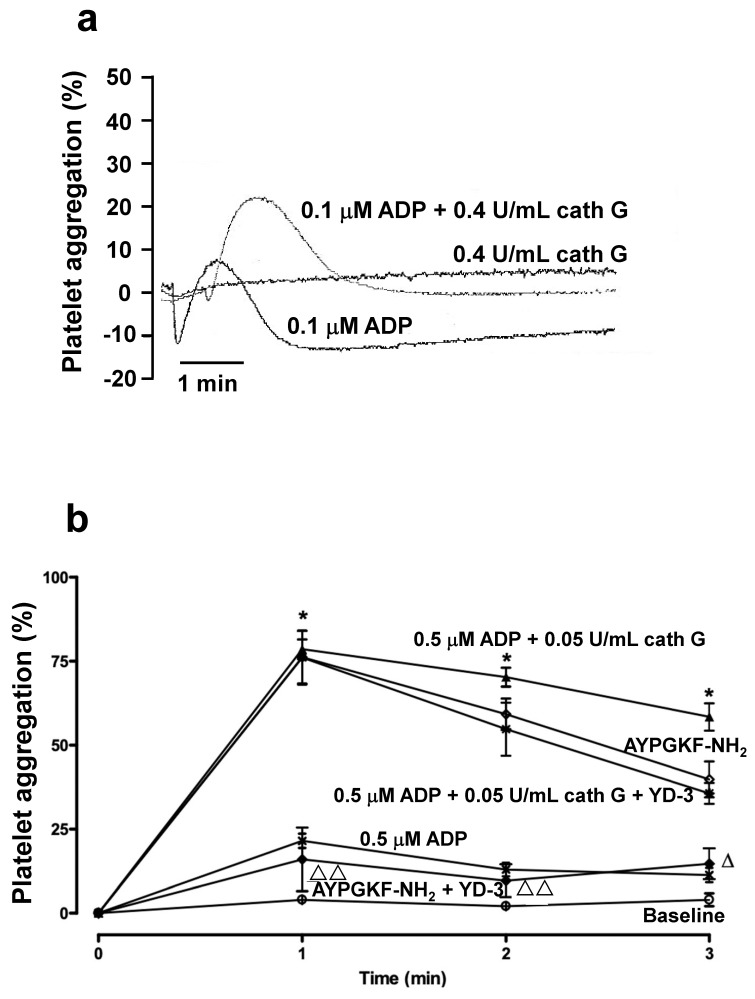
Murine platelet aggregation by cathepsin G: Need for blood cells beyond platelets. (**a**) Platelet aggregation tracings in platelet-rich plasma of Swiss WT mice, by 0.1 µM ADP, with or without cathepsin G (0.4 U/mL) pretreatment for 6 min at 37 °C, and by cathepsin G alone, as indicated. Continuous tracings recorded on a Chrono-log aggregometer are representative of 3 independent experiments. (**b**) Platelet aggregation curves for Swiss WT mouse blood, in the absence of added agonist (Baseline, open circles), or induced by 0.5 µM ADP without or with cathepsin G (0.05 U/mL) pretreatment for 6 min at 37 °C, in the absence or presence of 100 µM PAR4 antagonist YD-3; tracings (without ADP) induced by 30 µM PAR4 agonist AYPGKF-NH_2_ in the absence or presence of 100 µM PAR4 antagonist YD-3, as indicated. Data in **b** represent mean ± SEM (*n* = 3–13 in each group). Statistical analysis was by one-way ANOVA, followed by Newman–Keuls multiple-comparison test (* *p* < 0.0001 vs. tracing for 0.5 µM ADP; ^Δ^ *p* < 0.01, ^ΔΔ^ *p* < 0.0001 vs. tracing for AYPGKF-NH_2_). Platelet aggregation is calculated from residual platelet numbers at the indicated time points.

Nonetheless, in platelet-rich plasma from normal Swiss mice, cathepsin G failed to induce aggregation, as measured during classical aggregometry, even at high concentrations (0.4 U/mL) (Figure 4a). 

In contrast, low concentrations of ADP (0.1 µM) resulted in small but reversible platelet aggregation, indicative of weak and reversible platelet activation. Combining both ADP (0.1 µM) and 0.4 U/mL cathepsin G still proved to be inadequate (Figure 4a), findings in contrast with those shown in Figure 1 and Figure 2 for washed human platelets and in Figure 3 for whole blood. Rotating whole blood from Swiss mice in microtiter plates did not trigger platelet aggregation over a 3 min interval, in the absence of further platelet agonists, whereas weak and reversible mouse platelet aggregation was observed in the presence of 0.5 µM ADP (Figure 4b). However, mouse platelets in whole blood were potently aggregated in the presence of the PAR-4 agonist peptide AYPGKF-NH_2_, an aggregation which was abolished in the presence of the PAR4 antagonist YD-3 (Figure 4b).

**Figure 5:**
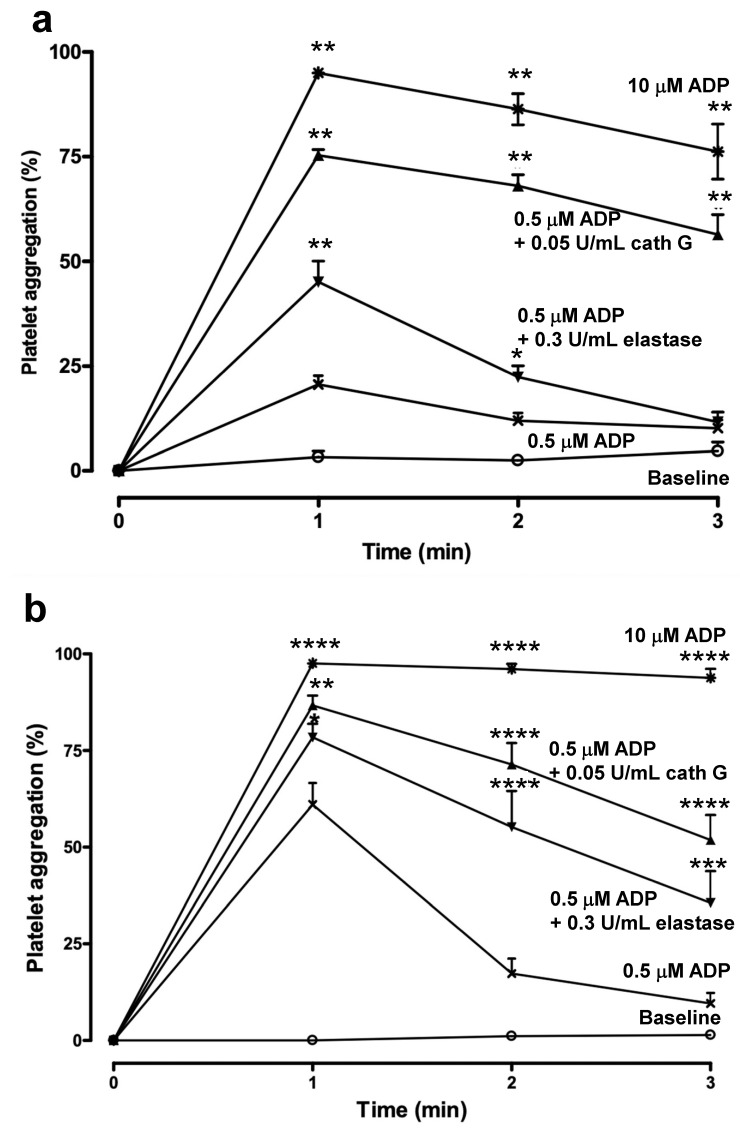
Whole blood aggregation in C57BL/6 WT vs. C57BL/6 *PAR4*^−/−^mice. (**a**) Platelet aggregation curves for C57BL/6 WT (**a**) and C57BL/6 *PAR4*^−/−^ mouse blood (**b**), in the absence of added agonist (Baseline, open circles), or upon addition of 0.5 µM ADP, alone or associated with 0.05 U/mL cathepsin or 0.3 U/mL elastase G, added during a 3 min pretreatment a 37 °C; the tracing for 10 µM ADP is also shown. Data are mean ± SEM (*n* = 4–12 in each group). Statistical analysis was by one-way ANOVA, followed by Newman–Keuls multiple-comparison test ((**a**) * *p* < 0.01, ** *p* < 0.0001 vs. tracing for 0.5 µM ADP; (**b**) * *p* < 0.05, ** *p* < 0.01, *** *p* < 0.001, **** *p* < 0.0001 vs. tracing for 0.5 µM ADP). Platelet aggregation is calculated from residual platelet numbers at the indicated time point.

In addition, the weak aggregation by 0.5 µM ADP was potentiated by 0.05 U/mL cathepsin G, as such was the case for human platelets (Figure 3) and this aggregation was not inhibited by the PAR-4 agonist peptide AYPGKF-NH_2_ (Figure 4b). Hence, to further document that also in mouse blood, platelet activation occurred via ADP and its receptors and not via PARs, we directly compared whole blood aggregation in C57BL/6 WT (Figure 5a) and C57BL/6 *PAR4*^−/−^ (Figure 5b) mice, genetically excluding contributions by PAR4 in platelet activation. 

Very similar aggregation profiles are shown in Figure 5a,b, which illustrate that (1) 0.3 U/mL elastase potentiates platelet aggregation mildly and reversibly and (2) 0.05 U/mL cathepsin G is at least as potent in stimulating platelet activation, (3) the activation profiles are not affected by the gene deficiency of PAR4 and (4) the overall platelet reactivity, as measured with 10 µM ADP is comparable for WT and *PAR4*^−/−^ mice (Figure 5).

**Figure 6:**
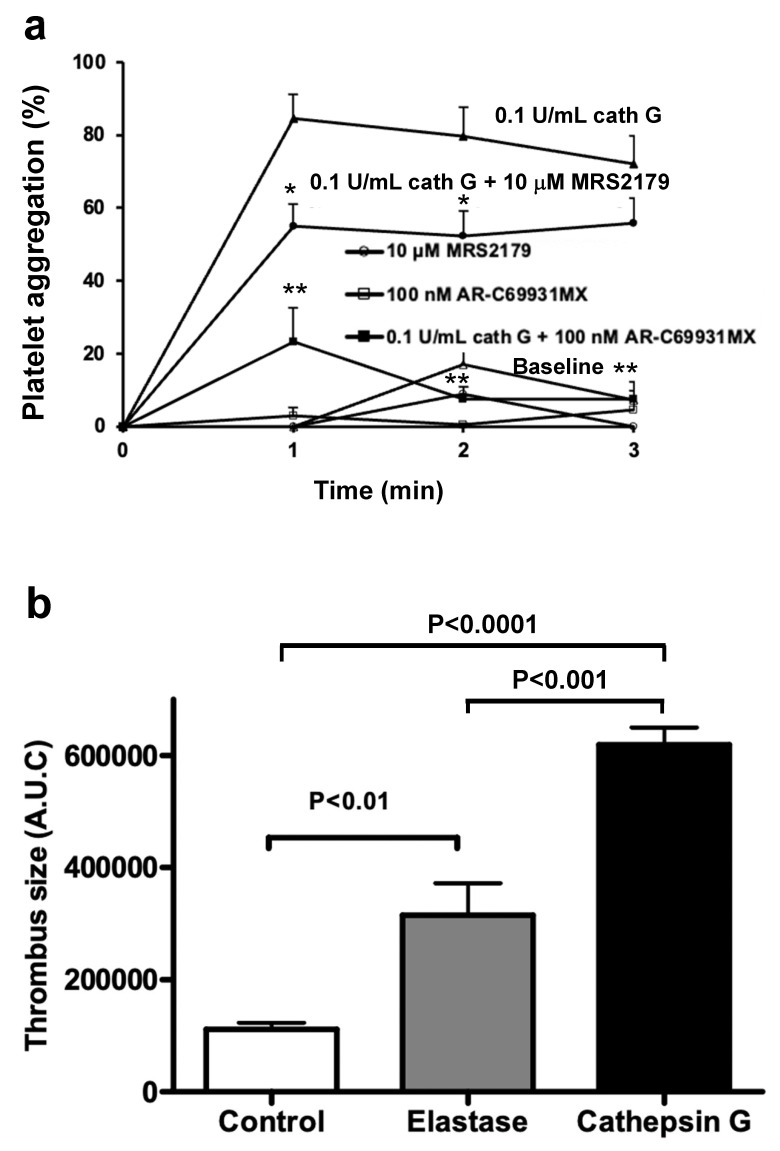
Murine platelet activation by elastase and cathepsin G in vitro and in vivo during experimental arterial thrombosis. (**a**) Platelet aggregation curves for Swiss WT mouse blood, in the absence of added agonist (Baseline, open triangles), or induced by 0.1 U/mL cathepsin G, in the absence or presence of the P2Y_1_ antagonist MRS2179 or the P2Y_12_ antagonist AR-C69931MX at the indicated concentrations; tracings without cathepsin G but supplemented with MRS2179 or AR-C69931MX are also shown. Platelet aggregation is calculated from residual platelet numbers at the indicated time points. (**b**) Cumulative thrombus size, expressed as total light intensity measured during 40 min (in arbitrary units [A.U.C.]), after a mild photochemical damage to the endothelium of the left carotid artery, in control Swiss mice and in mice pretreated with an i.v. bolus of elastase (24 U/kg) or cathepsin G (4 U/kg), administered 10 min before induction of photochemical injury. Data are mean ± SEM ((**a**) *n* = 3–5 in each group; (**b**) *n* = 4–6 in each group). Statistical analysis was by one-way ANOVA, followed by Newman–Keuls multiple-comparison test ((**a**) * *p* < 0.01, ** *p* < 0.0001 vs. tracing for 0.1 U/mL cathepsin G).

Switching back to Swiss mice and raising the cathepsin G concentration to 0.1 U/mL in the absence of added ADP confirmed the occurrence of potent platelet aggregation in murine whole blood, in the absence of further agonists (Figure 6a). The P2Y_1_ antagonist MRS2179 (10 µM) slightly reduced the cathepsin G-induced platelet aggregation and the P2Y_12_ antagonist AR-C69931MX (100 nM) abrogated it (Figure 6a). These findings clarified that, also in mouse blood, human cathepsin G triggers ADP-release from blood cells, other than platelets, a phenomenon responsible for P2 receptor mediated platelet activation and aggregation.

### 2.5. Cathepsin G in a Mouse Model of Arterial Thrombosis

Figure 6b illustrates, 10–15 min after injection of the PMN-derived inflammatory enzymes elastase or cathepsin G into the mouse circulation, that a prothrombotic tendency developed, measured as an increase in the cumulative mass of thrombus formed in vivo over the next 40 min, in the photochemically injured carotid artery. At a dose of 24 U/kg (targeting an initial plasma concentration of 0.3 U/mL), elastase enhanced arterial thrombosis 2.8-fold (*p* < 0.01), but cathepsin G, at a dose as low as 4 U/kg (targeting an initial plasma concentration of 0.05 U/mL), even promoted thrombosis to a greater extent (i.e., 5.5-fold (*p* < 0.001)).

## 3. Discussion

In the present study, the relationship between tissue inflammation and thrombosis was investigated in vivo and in vitro, via studying platelet activation by the neutrophil-released proteases cathepsin G and elastase. Depending on the concentration used, cathepsin G activated washed human platelets via PAR-1 or PAR-4, but added to murine or human whole blood at low concentrations, it potently activated platelets via an ADP-mediated mechanism, independently of PAR stimulation. This activation potently enhanced the thrombogenicity of arteries upon mild local endothelial damage, a condition accompanied by local and systemic inflammation [30].

Cathepsin G is long known to be a strong platelet agonist that triggers platelet aggregation between 25–200 nM [31] and plasma concentrations a high as 240 nM have been described after neutrophil activation [32]. The mean concentration of cathepsin G in normal dog plasma was determined to be 38 µg/mL, however primarily measured as cathepsin G/alpha 1-proteinase inhibitor complex [33]. The range of cathepsin G concentrations in this study (0.05–035 U/mL, corresponding to 0.83–5.83 µg/mL or 35–250 nM) agrees well with the range of concentrations that covers weak to strong inflammation and corresponding responses of platelets. Human platelets express PAR1 and PAR4 [25], whereas murine platelets express PAR3 and PAR4 [34]. In human platelets PAR1 is activated by low thrombin concentrations and PAR4 requires higher concentrations [25]. In murine platelets, PAR3 is not directly involved in platelet activation by thrombin; it cooperatively binds thrombin to its anionic binding site and increases the local thrombin concentration on the platelet surface, facilitating activation of PAR4 by thrombin [34]. Although PAR1 activation by cathepsin G has been reported via cleavage at the thrombin cleavage site between the N-terminal Arg^41^ and S^42^, the additional cleavage between F^56^ and W^57^ is believed to eliminate the new S^42^FLLRN^47^ N-terminus, required for PAR activation [26]. Our present findings with washed platelets demonstrate that low concentrations of cathepsin G can sensitize platelets via PAR1, without involving PAR4, the main determinant of platelet activation by higher cathepsin G concentrations. This suggests that in secluded micro-environments with only a few erythrocytes present, low concentrations of cathepsin G can trigger platelet activation via PAR1, in conjunction with other platelet agonists, thus amplifying thrombo-inflammation via platelet-leukocyte heteroconjugate formation and NETosis.

To our surprise, during whole blood aggregation experiments, we found no evidence of PAR involvement in platelet activation, neither in human blood, Swiss mouse blood, C57BL/6 WT mouse blood or C57BL/6 *PAR4*^−/−^ blood. No inhibitory effect was observed during platelet activation by low cathepsin G concentrations by selective antagonists of PAR1 or PAR4. The mildly higher platelet reactivity to 0.5 µM ADP in *PAR4*^−/−^ mouse platelets compared to WT platelets may reflect a gain in sensitivity to ADP, via the Gq coupled P2Y_1_ receptor, upon reorientation of that fraction of Gq, normally coupled to PAR4. This, however, was not further investigated.

Inhibition experiments in the presence of P2 receptor antagonists underscored that cathepsin G (0.1 U/mL) triggered platelet aggregation in whole blood, predominantly in an ADP-dependent fashion and primarily via platelet P2Y_12_. Yet, when tested at even higher concentrations, cathepsin G failed to induce aggregation in platelet-rich plasma, ruling out platelets to be the source of ADP in blood. This implicates that cathepsin G stimulated murine platelet activation indirectly, by triggering ADP release from other blood cells. Erythrocytes are the most likely candidate, as red blood cells play an important (also rheological) role in hemostasis. Prolonged bleeding times in anemia are corrected upon normalizing the hematocrit [35] and intact erythrocytes enhance the collagen-induced platelet responsiveness, via mechanisms that include the release of ADP [36]. Moreover, cathepsin G digests red blood cell membrane proteins, such as glycophorin A, B and C [37,38], resulting in cell membrane damage, facilitating the leakage of ADP from these cells. This interpretation is supported by the observation that anti-hemolytic drugs, such as chlorpromazine prolong the bleeding time by decreasing plasma ADP-levels [39]. Additionally, the more recent finding that intravascular hemolysis triggers ADP-mediated generation of platelet-rich thrombi in precapillary pulmonary arteries identify red blood cells as the source of ADP, released in sufficiently high concentrations to have prothrombotic consequences. Furthermore, platelet activation due to shearing of RBCs is reduced in the presence of apyrase which metabolizes ADP to AMP [40]. All this implies that the major cathepsin G substrate in blood consists not of platelets but red blood cells, which upon membrane proteolysis release ADP, further activating platelets, independently of PAR receptor activation. Because ADP is an agonist also involved in the later stages of thrombus maintenance, its long-lasting release from erythrocytes in inflammation (several hours to days) will counteract thrombus dissolution and sustain air pollutant-induced thrombotic complications. We cannot exclude that the proteolytic efficiency of human and murine neutrophil released proteases on red blood cell targets would be different, hence affecting the extent of ADP release and platelet sensitization differently in man and mouse. Moreover, we observe slight differences in potency for the P2 antagonists used in human and mouse blood. Nevertheless, P2 receptors are crucial both for human and mouse platelets and we found a clear parallel for the impact of human cathepsin G/elastase in human and murine blood in vitro. Hence, we believe that our findings in the mouse model in vivo are representative for the fate of platelets in man during exposure to air-pollution. Therefore, our findings advocate preventive use of P2 receptor antagonists (e.g., during forest fires) to protect those exposed to fire-generated particulate matter from increased potentially lethal particulate-induced thrombogenicity.

We found that cathepsin G enhances platelet activation in vivo and in vitro very similarly, at concentrations too low to trigger clear platelet activation directly. In combination with threshold concentrations of established platelets agonists, such as collagen and ADP, cathepsin G (0.05 U/mL) was slightly more potent than was elastase (0.3 U/mL) during in vitro whole blood aggregation assays. Likewise, in combination with the vascular ligands exposed to the circulation upon experimental carotid artery damage induction in vivo, cathepsin G was also more potent than elastase, when administered at doses, intended to result in similar concentrations as those evaluated in vitro. The experimental thrombosis model in this study was chosen to be able to compare our present findings with historical results on the thrombogenicity analyzed after i.t. instillation of DEP: the thrombus volume previously measured 6–24 h post-administration of DEP was increased 7–8-fold over baseline thrombosis [9], a factor that compares very well with the factors presently determined for cathepsin G (5.5) and elastase (2.8), these factors having to be interpreted in an integrated manner, since both enzymes are simultaneously released upon neutrophil activation. The present work, therefore, complements earlier work on particulate matter-induced inflammation and its consequences. Whereas it was clear that inhalation of air pollutants triggers lung inflammation lasting for several days [9], extending to circulating leukocytes, it is now clear how PMN-released proteases transmit leukocyte activation to platelet sensitization in the peripheral circulation. It is, therefore, evident that in cardiovascular compromised persons (e.g., with coronary microfissures, activated endothelium, damaged vascular wall, inflamed blood surfaces), sensitized platelets will amplify existing cardiovascular risk factors, as a result of their adhesion and aggregation, processes promoting arterial thrombosis.

In conclusion, PMN-derived cathepsin G plays an important role in circulating platelet activation at those concentrations that are secreted from leukocytes during mild inflammation. Cathepsin G secreted into blood, induces platelet sensitization indirectly upon ADP release from erythrocytes after cathepsin G lyses erythrocyte membrane proteins, thus amplifying the thrombogenicity of existing minor vascular lesions throughout the inflammation episode.

## 4. Materials and Methods

### 4.1. Materials

The PMN-derived protease elastase (30 U/mg) was from Serva (Heidelberg, Germany) and cathepsin G from Sigma (St. Louis, MO, USA) or Calbiochem (Darmstadt, Germany). These preparations were trypsin-free, when tested with the chromogenic substrate Bz-Ile-Glu-Gly-Arg-p-nitroanilide (Chromogenix, Antwerp, Belgium). The PAR1 agonist peptide TRAP (SFLLRN-NH_2_) was purchased from Sigma, as was the PAR1 antagonist peptide 3-Mpr-FCha-Cha-RKPNDK-NH_2_ (Mpr-NH_2_). Likewise, the PAR4 agonist peptide AYPGKF-NH_2_ and the PAR4 antagonist peptide trans-cinnamoyl-YPGKF-NH_2_ (tcY-NH_2_) were from Sigma. The potent PAR1 antagonist RWJ-58259 [41] and PAR4 antagonist YD-3 [42] were synthesized by the Pierre Fabre Research Institute (Castres, France) and kindly provided by Dr. Bruno Legrand. The P2Y_12_ antagonist AR-C69931MX was a kind gift of Astra Zeneca R+D (Charnwood, UK). The P2Y_1_ antagonist MRS2179 and ADP were from Sigma, collagen was from Nycomed (Brussels, Belgium), CellFix was from Becton Dickinson (Franklin Lakes, NJ, USA) and the anti-P-selectin antibody CD62-PE was obtained from Dako (Glusdorp, Denmark). Male and female wild-type mice with Swiss and C57BL/6 genetic background were generated in the KU Leuven animal facility. C57BL/6 *PAR4*^−/−^ mice were kindly provided by Dr. S. Coughlin (San Francisco, CA, USA) and were bred into the C57BL/6 genetic background of the KU Leuven animal facility for at least 4 generations.

### 4.2. Unit Definition for Cathepsin G

One U/mL cathepsin G was defined as the concentration of enzyme that releases one nanomole/mL.sec from N-succinyl-Ala-Ala-Pro-Phe-*p*-nitroanilide (Chromogenix, Antwerp, Belgium), at pH 7.5 and 37 °C. According to this definition, human neutrophil cathepsin G from Calbiochem (95% pure by SDS-PAGE) had a specific activity of 35 U/mg, compared to 60 U/mg for the purified human leukocyte cathepsin G from Sigma.

### 4.3. Blood Collection, Plasma Isolation and Platelet Preparation

Murine blood was collected from mice, anesthetized with Nembutal (60 mg/kg i.p.), by puncture of the retro-orbital sinus and freely drained into 3.2% citrate. Male and female WT and *PAR4*^−/−^ C57BL/6 mice weighing 20–25 g were bled for the analysis of PAR4 contributions in the cathepsin G induced platelet aggregation, whereas Swiss mouse blood was used for all other aggregation experiments in whole blood and in platelet-rich plasma.

Human platelets were isolated from blood from healthy volunteers by puncture of the antecubital vein and freely drained in acid-citrate-dextrose (ACD; 93 mM sodium citrate, 7 mM citric acid, 0.14 mM dextrose, pH 6.5) in a volume ratio of ACD to blood of 1:6, in the presence of 0.5 U/mL apyrase. Platelet-rich plasma was obtained by centrifugation at 150× *g* for 15 min, after which the PRP was diluted 3-fold in ACD (pH 6.5) and centrifuged once more for 10 min at 800× *g* in order to obtain washed platelets. The platelet pellet was resuspended in Tyrode buffer (137 mM NaCl, 12 mM NaHCO_3_, 2.7 mM KCl, 0.43 mM Na_2_HPO_4_, 1 mM MgCl_2_, 5.5 mM glucose, and 5 mM HEPES, pH 7.3), at a density of 3.0 × 10^5^ platelets/µL.

### 4.4. Platelet Aggregation in Murine and Human Whole Blood

Blood, withdrawn as described above, was aliquoted at 250 µL in the wells of a 48 well plate, positioned in a Thermostar at 37 °C (BMG LABTECH GmbH, Offenburg, Germany). Blood was pretreated with the indicated antagonist, prior to addition of elastase (0.3 U/mL) or cathepsin G (0.05 or 0.1 U/mL) for an additional 2–6 min without shaking, as further specified per experiment. Next, the plate was rotated and brought to 900 rpm over a 1 min interval. When indicated, for the WT and *PAR4*^−/−^ C57BL/6 mouse platelet aggregations, ADP (0.5 µM) was then added to initiate aggregation. At different time points, 25 µL samples were removed and fixed on ice in 225 µL cellFix for 1 h. After fixation, single platelets were counted in a Cell-Dyn 3500 (Mountain View, CA USA). The percentage aggregation is defined as the difference between the number of platelets present at the time of blood collection and the number of single remaining platelets, at each time point, expressed as a percentage of the initial platelet number. Thus, 100% aggregation represents a residual platelet count equal to zero.

### 4.5. Washed Human Platelet Aggregation and Mouse PRP Aggregation

Washed human platelet aggregation was performed on a dual-channel Chrono-Log Aggregometer (ChronologCorp, Havertown, PA, USA). Thus, 100% aggregation represents the difference in light transmission between the platelet suspension and that of the suspension buffer, containing 10% human plasma, because at complete platelet aggregation, light diffraction by suspended platelets has disappeared. Platelet aggregation by collagen (0.1–0.25 μg/mL) was investigated as a function of platelet pretreatment with increasing concentrations of elastase (0.05–0.3 U/mL) or cathepsin G (0.05–0.4 U/mL). Elastase or cathepsin G were added to platelets, resuspended to 300,000/μL for 6 min, at 37 °C before induction of stirring and administration of collagen or ADP. Plasma (final concentration 10%) was added together with platelets agonists, as a source of fibrinogen, to facilitate platelet aggregation. To assess the role of PAR receptors, platelets were pretreated with the PAR1 receptor antagonist 3-Mpr-FCha-Cha-RKPNDK-NH_2_ (Mpr-NH_2_, Sigma, St. Louis, MO, USA) or RWJ-58259, or the PAR4 receptor antagonist trans-cinnamoyl-YPGKF-NH_2_ (tcY-NH_2_; Sigma) or YD-3, prior to addition of elastase, cathepsin G, and/or the platelet agonists collagen or ADP. For experiments at higher cathepsin G concentrations (0.35 U/mL), inducing platelet aggregation by itself, collagen was omitted. The presence of trypsin in the elastase preparation was tested with the chromogenic substrate Bz-Ile-Glu-Gly-Arg-p-nitroanilide (Chromogenix, Antwerp, Belgium), in comparison to elastase substrate N-methoxy succinyl-Ala-Ala-Pro-Val p-nitroanilide (Chromogenix, Antwerp, Belgium). No absorbance (405 nm) was generated with Bz-Ile-Glu-Gly-Arg-p-nitroanilide, after 24 h (not shown).

Mouse platelet-rich plasma (PRP) was aggregated in the dual-channel Chrono-Log Aggregometer, where it was stirred at 1000 RPM. Aggregation at time 0 was triggered with ADP, cathepsin G or their mixture, at the indicated concentrations. Here, 100% aggregation represents the difference in light transmission between PRP and mouse plasma, because at complete platelet aggregation, light diffraction by suspended platelets has disappeared.

### 4.6. Detection by Flow Cytometry of Activated Platelets Expressing P-Selectin (CD62)

In addition to platelet aggregation, the elastase and cathepsin G mediated platelet activation was studied by detection of the platelet activation marker P-selectin (CD62), via flow cytometry. For this purpose, 25 μL of washed human platelets (300,000/μL) were incubated for 1 min at 37 °C with 2 mM CaCl_2_ and, when indicated, with 10 μM 3-Mpr-FCha-Cha-RKPNDK-NH_2_ or tcY-NH_2_. Elastase (0.3 U/mL) and/or cathepsin G (0.05 U/mL) were then added, and incubated for 6 min at 37 °C. Thereafter, the samples were centrifuged for 2 min at 13,000 rpm, and the pellet resuspended in 30 μL of Tyrode buffer with 2 mM CaCl_2_ and the reaction was stopped by addition of 315 μL of paraformaldehyde (1%), for 1 h, at 4 °C. As a positive control, convulxin (15 ng/mL, Sigma, a snake-venom protein, capable of GPVI activation) was added to separate tubes, for 5 min at 37 °C, after which samples were fixed with paraformaldehyde. Samples were then centrifuged (13,200 rpm, for 10 min), and the pellet washed with PBS. Upon re-centrifugation for 10 min at 13,200 rpm, the pellet was resuspended in 50 μL PBS, supplemented with CD62-PE (Dako, Glusdorp, Denmark) and incubated for 20 min at room temperature. Then 1 mL of PBS was added and sample acquisition on a FACSCalibur (Becton and Dickinson, San José, CA, USA) was done immediately.

### 4.7. Western Blot Analyses

Aliquots of human or Swiss mouse washed platelets (25 μL) were incubated with 2mM CaCl_2_ and elastase or cathepsin G, with or without pretreatment with 3-Mpr-FCha-Cha-RKPNDK-NH_2_ or tcY-NH_2_. The thrombin receptor agonist peptide TRAP (SFLLRN; Bachem, Weil am Rhein, Germany) was used as a positive control for PAR1 receptor activation, at 10 μM. In addition, the PAR4 receptor agonist peptide AYPGKF-NH_2_ was used at 30 μM. Washed murine or human platelets were incubated without stirring for 3 min at 37 °C, upon which samples were centrifuged for 10 min at 13,200 rpm. Platelet pellet was then dissolved by adding 15 μL of sodium dodecyl sulfate (SDS) sample buffer (62.5 mM Tris, 2% SDS, 10% glycerol, 0.01% bromphenol blue), 50 μM dithiothreitol (DTT), 100 μM NaF and 2 μM Na_2_CO_3_. Samples were boiled at 95 °C for 5 min and loaded on a 10% SDS-PAGE gel. Alternatively, untreated mouse platelets were incubated for 3 min at 37 °C, after which 12 μL of 4× concentrated SDS sample buffer was added (without centrifugation), upon which samples were boiled and subjected to SDS-PAGE. After electrophoretic transferal of the proteins to nitrocellulose, membranes were blocked in Tris-buffered saline-Tween-milk buffer and incubated overnight at 4 °C with the anti-phosphotyrosine mAb 4G10 (Bioconnect, Huissen, The Netherlands). After adding horseradish peroxidase-conjugated polyclonal goat anti-mouse antibody GAM-PO (DakoCytomation, Glostrup, Denmark), immunoreactive bands were visualized by the enhanced chemiluminescence system (Amersham Biosciences, Uppsala, Sweden). Phosphorylation of phospholipase Cβ2 can be identified at a specific position on the gel, even in the presence of other tyrosine-phosphorylated platelet protein bands.

### 4.8. Experimental Arterial Thrombosis Model

Male and female Swiss mice weighing 45–50 g were used. Upon anesthesia with sodium pentobarbital (60 mg/kg, i.p.), they were placed in a supine position on a heating pad at 37 °C, tracheotomized, and artificially ventilated (Hugo Sachs Apparatus Minivent type 845 respirator; Hugo Sachs Elektronik-Harvard Apparatus GmbH, March-Hugstetten, Germany). A 2F venous catheter (Portex, Hythe, U.K.) was inserted in the right jugular vein for the administration of Rose Bengal (Sigma) and elastase or cathepsin G. The right carotid artery was exposed from the surrounding tissue and mounted on a house-made transilluminator. Ten min after intravenous administration of elastase or cathepsin G, Rose Bengal (20 mg/kg) was injected and a segment of the carotid artery was irradiated with green light (540 nm) for 90 s, using an optic fiber mounted on a micromanipulator located 5 mm above the artery. The developing thrombus was monitored under a microscope at 40× magnification. The change over time in light transmission through the blood vessel at the site of the trauma was recorded using a microscope-attached camera. Images were recorded at intervals of 10 s over a time period of 40 min. Image analysis was used to quantitate thrombus intensity. The size of the thrombus was expressed in arbitrary units (A.U.) as the total area under the curve (A.U.C.), when plotting light intensity against time [43]. The mice were euthanized at the end of the recording.

### 4.9. Statistics

Data are expressed as means ± SEM. Comparisons between groups were performed by one way analysis of variance (ANOVA), followed by Newman–Keuls multiple-range tests. *p*-values less than 0.05 were considered to be significant.

## Data Availability

Original data are available as Excel sheets and additional preliminary figures, as well as complementary analyses and text draft.

## References

[1] Brook R.D., Rajagopalan S., Pope C.A., Brook J.R., Bhatnagar A., Diez-Roux A.V., Holguin F., Hong Y., Luepker R.V., Mittleman M.A. (2010). American Heart Association Council on Epidemiology and Prevention, Council on the Kidney in Cardiovascular Disease, and Council on Nutrition, Physical Activity and Metabolism. Particulate matter air pollution and cardiovascular disease: An update to the scientific statement from the American Heart Association. Circulation.

[2] Franklin B.A., Brook R., Pope C.A. (2015). Air pollution and cardiovascular disease. Curr. Probl. Cardiol..

[3] Newby D.E., Mannucci P.M., Tell G.S., Baccarelli A.A., Brook R.D., Donaldson K., Forastiere F., Franchini M., Franco O.H., Graham I. (2015). ESC Working Group on Thrombosis, European Association for Cardiovascular Prevention and Rehabilitation; ESC Heart Failure Association. Expert position paper on air pollution and cardiovascular disease. Eur. Heart J..

[4] Hennig F., Geisel M.H., Kälsch H., Lucht S., Mahabadi A.A., Moebus S., Erbel R., Lehmann N., Jöckel K.H., Scherag A. (2020). Heinz Nixdorf Recall Study Investigative Group. Air Pollution and Progression of Atherosclerosis in Different Vessel Beds-Results from a Prospective Cohort Study in the Ruhr Area, Germany. Environ. Health Perspect..

[5] Jacobs L., Emmerechts J., Hoylaerts M.F., Mathieu C., Hoet P.H., Nemery B., Nawrot T.S. (2016). Traffic air pollution and oxidized LDL. PLoS ONE.

[6] Nemmar A., Hoet P.H., Dinsdale D., Vermylen J., Hoylaerts M.F., Nemery B. (2003). Diesel exhaust particles in lung acutely enhance experimental peripheral thrombosis. Circulation.

[7] Nemmar A., Vanbilloen H., Hoylaerts M.F., Hoet P.H., Verbruggen A., Nemery B. (2001). Passage of intratracheally instilled ultrafine particles from the lung into the systemic circulation in hamster. Am. J. Respir. Crit. Care Med..

[8] Nemmar A., Hoet P.H., Vanquickenborne B., Dinsdale D., Thomeer M., Hoylaerts M.F., Vanbilloen H., Mortelmans L., Nemery B. (2002). Passage of inhaled particles into the blood circulation in humans. Circulation.

[9] Nemmar A., Nemery B., Hoet P.H., Vermylen J., Hoylaerts M.F. (2003). Pulmonary inflammation and thrombogenicity caused by diesel particles in hamsters: Role of histamine. Am. J. Respir. Crit. Care Med..

[10] Nemmar A., Hoylaerts M.F., Hoet P.H., Vermylen J., Nemery B. (2003). Size effect of intratracheally instilled particles on pulmonary inflammation and vascular thrombosis. Toxicol. Appl. Pharmacol..

[11] Burger P.C., Wagner D.D. (2003). Platelet P-selectin facilitates atherosclerotic lesion development. Platelet P-selectin facilitates atherosclerotic lesion development. Blood.

[12] Theilmeier G., Lenaerts T., Remacle C., Collen D., Vermylen J., Hoylaerts M.F. (1999). Circulating activated platelets assist THP-1 monocytoid/endothelial cellinteraction under shear stress. Blood.

[13] Nemmar A., Hoet P.H., Vandervoort P., Dinsdale D., Nemery B., Hoylaerts M.F. (2007). Enhanced peripheral thrombogenicity after lung inflammation is mediated by platelet-leukocyte activation: Role of P-selectin. J. Thromb. Haemost..

[14] Steinhoff M., Buddenkotte J., Shpacovitch V., Rattenholl A., Moormann C., Vergnolle N., Luger T.A., Hollenberg M.D. (2005). Proteinase-activated receptors: Transducers of proteinase-mediated signaling in inflammation and immune response. Endocr. Rev..

[15] Rabhi-Sabile S., de Romeuf C., Pidard D. (1998). On the mechanism of plasmin-induced aggregation of human platelets: Implication of secreted von Willebrand factor. Thromb. Haemost..

[16] Quinton T.M., Kim S., Derian C.K., Jin J., Kunapuli S.P. (2004). Plasmin-mediated activation of platelets occurs by cleavage of protease-activated receptor 4. J. Biol. Chem..

[17] Asfaha S., Cenac N., Houle S., Altier C., Papez M.D., Nguyen C., Steinhoff M., Chapman K., Zamponi G.W., Vergnolle N. (2007). Protease-activated receptor-4: A novel mechanism of inflammatory pain modulation. Br. J. Pharmacol..

[18] Cottrell G.S., Amadesi S., Grady E.F., Bunnett N.W. (2004). Trypsin IV, a novel agonist of protease-activated receptors 2 and 4. J. Biol. Chem..

[19] Renesto P., Chignard M. (1995). Neutrophil-mediated platelet activation: A key role for serine proteinases. Gen. Pharmacol..

[20] Selak M.A., Chignard M., Smith J.B. (1988). Cathepsin G is a strong platelet agonist released by neutrophils. Biochem. J..

[21] Si-Tahar M., Pidard D., Balloy V., Moniatte M., Kieffer N., Van Dorsselaer A., Chignard M. (1997). Human neutrophil elastase proteolytically activates the platelet integrin alphaIIbbeta3 through cleavage of the carboxyl terminus of the alphaIIb subunit heavy chain. Involvement in the potentiation of platelet aggregation. J. Biol. Chem..

[22] Selak M.A. (1992). Neutrophil elastase potentiates cathepsin G-induced platelet activation. Thromb. Haemost..

[23] Evangelista V., Piccardoni P., White J.G., de Gaetano G., Cerletti C. (1993). Cathepsin G-dependent platelet stimulation by activated polymorphonuclear leukocytes and its inhibition by antiproteinases: Role of P-selectin-mediated cell–cell adhesion. Blood.

[24] Evangelista V., Rajtar G., de Gaetano G., White J.G., Cerletti C. (1991). Platelet activation by fMLP-stimulated polymorphonuclear leukocytes: The activity of cathepsin G is not prevented by antiproteinases. Blood.

[25] Sambrano G.R., Huang W., Faruqi T., Mahrus S., Craik C., Coughlin S.R. (2000). Cathepsin G activates protease-activated receptor-4 in human platelets. J. Biol. Chem..

[26] Molino M., Blanchard N., Belmonte E., Tarver A.P., Abrams C., Hoxie J.A., Cerletti C., Brass L.F. (1995). Proteolysis of the human platelet and endothelial cell thrombin receptor by neutrophil-derived cathepsin G. J. Biol. Chem..

[27] Si-Tahar M., Renesto P., Falet H., Rendu F., Chignard M. (1996). The phospholipase C/protein kinase C pathway is involved in cathepsin G-induced human platelet activation: Comparison with thrombin. Biochem. J..

[28] Faraday N., Schunke K., Saleem S., Fu J., Wang B., Zhang J., Morrell C., Dore S. (2013). Cathepsin G-dependent modulation of platelet thrombus formation in vivo by blood neutrophils. PLoS ONE.

[29] Leger A.J., Jacques S.L., Badar J., Kaneider N.C., Derian C.K., Andrade-Gordon P., Covic L., Kuliopulos A. (2006). Blocking the protease-activated receptor 1–4 heterodimer in platelet-mediated thrombosis. Circulation.

[30] Franchini M., Veneri D., Lippi G. (2007). Inflammation and hemostasis: A bidirectional interaction. Clin. Lab..

[31] Molino M., Lallo M.D., de Gaetano G., Cerletti C. (1992). Intracellular Ca^2+^ rise in human platelets induced by polymorphonuclear-leucocyte-derived cathepsin G. Biochem. J..

[32] Perrin J., Lecompte T., Tournier A., Morlon L., Marchand-Arvier M., Vigneron C. (2010). In vitro effects of human neutrophil cathepsin G on thrombin generation: Both acceleration and decreased potential. Thromb. Haemost..

[33] Björk P., Axelsson L., Ohlsson K. (1991). Release of dog polymorphonuclear leukocyte cathepsin G, normally and in endotoxin and pancreatitic shock. Isolation and partial characterization of dog polymorphonuclear leukocyte cathepsin G. Biol. Chem. Hoppe Seyler.

[34] Nakanishi-Matsui M., Zheng Y.W., Sulciner D.J., Weiss E.J., Ludeman M.J., Coughlin S.R. (2000). PAR3 is a cofactor for PAR4 activation by thrombin. Nature.

[35] Anand A., Feffer S.E. (1994). Hematocrit and bleeding time: An update. South. Med. J..

[36] Valles J., Santos M.T., Aznar J., Marcus A.J., Martinez-Sales V., Portoles M., Broekman M.J., Safier L.B. (1991). Erythrocytes metabolically enhance collagen-induced platelet responsiveness via increased thromboxane production, adenosine diphosphate release, and recruitment. Blood.

[37] Bykowska K., Duk M., Kusnierz-Alejska G., Kopec M., Lisowska E. (1993). Degradation of human erythrocyte surface components by human neutrophil elastase and cathepsin G: Preferential digestion of glycophorins. Br. J. Haematol..

[38] Bykowska K., Duk M., Kusnierz-Alejska G., Sikorska A., Letowska M., Mendek-Czajkowska E., Lopaciuk S., Kopec M., Lisowska E. (1997). Degradation of glycophorin A of human erythrocytes in patients with myelo-or lymphoproliferative disorders: Possible role of neutrophil proteases. Br. J. Haematol..

[39] Born G.V., Wehmeier A. (1979). Inhibition of platelet thrombus formation by chlorpromazine acting to diminish haemolysis. Nature.

[40] Helms C.C., Marvel M., Zhao W., Stahle M., Vest R., Kato G.J., Lee J.S., Christ G., Gladwin M.T., Hantgan R.R. (2013). Mechanisms of hemolysis-associated platelet activation. J. Thromb. Haemost..

[41] Damiano B.P., Derian C.K., Maryanoff B.E., Zhang H.C., Gordon P.A. (2003). RWJ-58259: A selective antagonist of protease activated receptor-1. Cardiovasc. Drug Rev..

[42] Wu C.C., Hwang T.L., Liao C.H., Kuo S.C., Lee F.Y., Lee C.Y., Teng C.M. (2002). Selective inhibition of protease-activated receptor 4-dependent platelet activation by YD-3. Thromb. Haemost..

[43] Stockmans F., Stassen J.M., Vermylen J., Hoylaerts M.F., Nystrom A. (1997). A technique to investigate mural thrombus formation in small arteries and veins: I. Comparative morphometric and histological analysis. Ann. Plast. Surg..

